# Novel micro-MRI approach for subchondral trabecular bone analysis in patients with hip osteoarthritis is comparable to micro-CT approach

**DOI:** 10.3325/cmj.2022.63.515

**Published:** 2022-12

**Authors:** Tea Duvančić, Siniša Škokić, Igor Erjavec, Mihovil Plečko, Ivan Bohaček, Srećko Gajović, Domagoj Delimar

**Affiliations:** 1 University of Zagreb School of Medicine, Zagreb, Croatia; 2 Laboratory for Regenerative Neuroscience – GlowLab, Croatian Institute for Brain Research, University of Zagreb School of Medicine, Zagreb, Croatia; 3 University Hospital Center Zagreb, Zagreb, Croatia; 4 Department of Orthopedic Surgery, University of Zagreb School of Medicine, University Hospital Center Zagreb, Zagreb, Croatia; The first two authors contributed equally.

## Abstract

**Aim:**

To test the agreement between a newly developed micro-magnetic resonance imaging (MRI) analysis of the subchondral bone and the micro-computed tomography (CT) approach.

**Methods:**

Samples obtained from 10 patients with osteoarthritis undergoing total hip arthroplasty were scanned with a 7.0 T micro-MRI. Proton density-weighted images and proton density-weighted images with fat suppression were obtained. The results were validated with a micro-CT device. Micro-MRI and micro-CT scans of the same sample were aligned, and regions of interest were delineated on equal areas of the sample. Bone volume fraction was calculated by using in-house plugins. The agreement between the methods was tested with Bland-Altman analysis.

**Results:**

The agreement between the methods was good, with average difference of 2.167%. The differences between the methods were not significant (*P* = 0.272, *t* test).

**Conclusion:**

The novel micro-MRI approach could be used for subchondral bone analysis. With further optimization for clinical MRI machines, the approach can be also used in the diagnostics of hip osteoarthritis.

Osteoarthritis (OA) is a progressive disorder that affects articular cartilage and subchondral bone. It is characterized by the development of subchondral cysts, bone marrow lesions, osteophytes, and a variety of other degenerative changes (1). The disorder is one of the leading causes of disability. Its prevalence is growing with obesity and age (2) – OA affects more than 60% of the population older than 60. However, it also affects younger people (3,4). It is a major economic burden, with a yearly cost of hip and knee replacements of £850 million in the United States alone (5). Although the available surgical treatments significantly improve the quality of life, no therapies are able to repair damaged joint tissue and to considerably postpone the need for surgical replacement. Recently, some progress has been made in terms of gene therapy and tissue engineering.

The increase in OA prevalence highlights the need for an effective therapy. If we want to understand OA progression we need to apply a holistic approach. Only by studying cartilage, the underlying subchondral bone, and the synovial membrane together can we gain a detailed insight into the triggers of degenerative changes. This insight can improve OA diagnostics and lead to the development of an effective OA therapy (1-4,6,7).

When it comes to OA studies, the main focus so far has been on cartilaginous tissue. Since articular cartilage has a high water content, magnetic resonance imaging (MRI) is the radiological method of choice for studying cartilage changes (8). Compositional MRI techniques, which visualize and quantify biochemical changes of the cartilage and other joint tissues, have revolutionized the imaging of articular joints. As MRI is able to detect biochemical changes before their morphological manifestation, it has markedly improved OA diagnostics and helped us to better understand the molecular background of OA (9-13). The degenerative changes of the subchondral bone induced by OA, on the other hand, are best visualized by computed tomography (CT) (14-17). Even though changes of subchondral bone play an important role in OA progression, they have not yet been studied in much detail. Researchers who prioritize MRI-based cartilage analysis over bone research mainly do so due to the overall emphasis on degenerative changes of cartilage in OA. Besides MRI, contrast-enhanced CT is also used to evaluate cartilage and provide information on collagen structure, proteoglycan content, and the severity of OA (18-21).

Although CT has some advantages over MRI, such as being cheaper and faster, its main disadvantages are exposure to ionizing radiation and lack of soft-tissue contrast. MRI, on the other hand, offers excellent soft-tissue contrast and is the method of choice for studying structural changes of articular cartilage (22). Given the MRI’s superior ability to visualize soft tissues, it is a better choice than CT for the whole-joint tissue analysis. Currently available MRI-based methods are mostly used for the analysis of bone marrow fat content and visualization of bone marrow lesions. These methods do not provide quantitative information on bone structure, such as bone volume fraction, which is often used in OA grading. Moreover, their reliance on complex sequences makes them not easily accessible (23-27).

A novel approach to trabecular bone structure analysis by micro-MRI allowed us to compare for the first time micro-MRI and micro-CT scans using ImageJ software. Our newly-developed ImageJ plugins can be used to acquire valuable quantitative information about trabecular bone structure, ie, bone volume percentage, as well as information about bone marrow fat composition. The validity of our new approach was tested by comparison with micro-CT – the gold standard in bone analysis. In addition to conventional MRI-based protocols used for studying cartilaginous tissue, by utilizing our novel approach the whole osteochondral unit can be studied using only one scanning method.

## Patients and methods

### Patients

Osteochondral tissue samples were obtained from patients with primary OA (n = 10; mean age 67.7 years; age range 43-86 years; 6 women) undergoing total hip arthroplasty (THA) at the Department of Orthopedic Surgery, University Hospital Center Zagreb, Croatia between May and July 2021. This study was approved by the Research Ethics Committees of University Hospital Center Zagreb and of the University of Zagreb School of Medicine. All patients gave informed consent to participate.

### Samples

Patients were assigned labels K-F1 to K-F10, and the samples were labeled accordingly. The samples were obtained intraoperatively from a femoral head, with a 10-mm diameter cylindrical chisel (Small Joint OATS Set, 10mm AR-8981-10S, Arthrex, Naples, FL, USA). They were stored in 15-mL polypropylene tubes filled with fresh normal saline solution (0.90% w/v of NaCl). Fixation in formaldehyde was omitted to avoid dehydration and to ensure better translation to clinical environment. Samples were scanned with a micro-MRI machine in the first free-time slot (within 24 h from the surgery), and with a micro-CT machine immediately afterwards.

### Micro-MR imaging of osteochondral tissue samples

An ultra-high-field (7 T) preclinical MRI scanner (BioSpec 70/20 USR, Bruker Biospin, Ettlingen, Germany) was used in a Tx/Rx configuration, with an 86-mm volume coil (MT0381, Bruker Biospin) for transmitting (Tx) and a two-element mouse brain surface coil (MT0042, Bruker Biospin) for receiving (Rx). Scans were acquired using Paravision software 6.0.1 (Bruker Biospin).

Explanted samples in 15-mL tubes were positioned as close as possible to the receive coil with the cartilage facing toward the coil to maximize the available signal. A water-filled capillary was taped to the outer edge of the tube in the sample area to serve as a guide for manual alignment (co-registration) of MRI images and micro-CT images, so that the same regions of interest (ROI) could be analyzed with both imaging techniques.

### Micro-CT imaging of osteochondral tissue samples

A micro-CT scanner (SkyScan 1076, Bruker, Kontich, Belgium) was used at an 18-μm isotropic voxel size with a voltage of 50 kV and an electric current of 140 μA. Beam-hardening was reduced using a 0.025-mm thick titanium filter. The rotational shift was set to 0.4° in an area of 198° for every sample, resulting in 495 projection images. The acquired data were reconstructed using the NRecon software (Bruker).

### MR image analysis and measurements

Bone volume and fat-water ratio were assessed with a pair of matched high-resolution Proton Density Turbo Spin-Echo scans. All scan parameters were identical, except the Fat Suppression module, which was turned off in the first and turned on in the second scan. Furthermore, the second scan was performed without automatic scan adjustments (reference power, receiver gain, etc) to prevent bias errors introduced by the automatic scan setup. The corresponding scan parameters were copied from the first scan.

Very long repetition time of 4000 ms was used to reach full magnetization recovery of both water and fat molecules, while echo time was set to the minimum allowed by the MRI system in the given conditions. The fat-shift artifact, caused by faster T2 relaxation of fat protons than of water protons, was limited to an acceptable amount of 1.5 px by increasing the receiver bandwidth to 750 Hz/px.

Bone percentage and fat fraction in the selected ROI were assessed with FIJI/ImageJ software (National Institutes of Health, Bethesda, MD, USA) via an in-house tool. Bone was separated from soft tissue using simple thresholding, whereby the threshold was determined from pilot scans. To minimize the dependence of measurements on fat content within the ROI, only the pixels darker than the set threshold in scans both with and without fat suppression were classified as bone. Bone volume percentage in the sample was then determined by assessing the fraction of the total ROI area covered in bone pixels across all selected slices.

Proton density fat fraction (PDFF) was calculated only on soft-tissue pixels, ie, on the inverse selection of “bone” pixels, using the expression FF = SIfat/SIfat + water. Here, SIfat + water represents all available MRI signal measured with the PD-weighted scan without fat suppression, while SIfat represents only the signal from fat molecules. SIfat is in fact a calculated image obtained by subtracting the fat-suppressed (ie, water-only) image (PDw-FS sequence) from the combined signal (both fat and water, PDw sequence). Calculating the ratio of these images yields a voxel-wise map of the fat fraction ranging from 0 to 1. The sample-wise average and standard deviation of measured fat fraction were calculated by applying the combined variance formulas, bearing in mind varying ROI size between slices and a different number of excluded bone pixels in each ROI (28-30).

### Micro-MRI and micro-CT scans alignment

Before bone volume calculations, we aligned micro-MRI and micro-CT scans to ensure the analysis of identical parts of the sample on both scans (Table 1). In this case, automatic co-registration tools fail to produce good alignment, not only because micro-CT and MRI differ in resolution and voxel shape but because they observe substantially different objects: native micro-CT does not detect soft tissues (eg, cartilage, fat), while native MRI does not detect hard tissues such as bones and always misregisters (displaces) fat tissue voxels to some degree due to the fat-shift artifact.

**Table 1:** General sequence of the steps in the process of micro-magnetic resonance imaging (MRI) and micro-computed tomography (CT) scans alignment and bone volume calculations, with a list of software and plugins used and a corresponding numerical label of each step

Step	Action	Software/plugin used
**1**	Alignment of a micro-CT scan with the capillary wall	DataViewer
**1b**	Resizing of a micro-CT scan	DataViewer
**2**	Alignment of micro-CT with a micro-MRI scan	ImageJ/Align images
**3**	Rotating a micro-CT scan to the same orientation as a micro-MRI scan	DataViewer
**3b**	Saving a micro-CT ROI* data set for a micro-CT analysis	CTAn
**3**	Saving a micro-CT ROI data set for ROI data set repositioning	CTAn
**4**	Adapting a micro-CT ROI set for micro-MRI	ImageJ/Adapt CT ROI to MRI, Align images
**5**	Micro-MRI bone volume calculation	ImageJ/Bone vs fat
**5**	Micro-CT bone volume calculation	CTAn

*ROI – region of interest.

**Figure 1:**
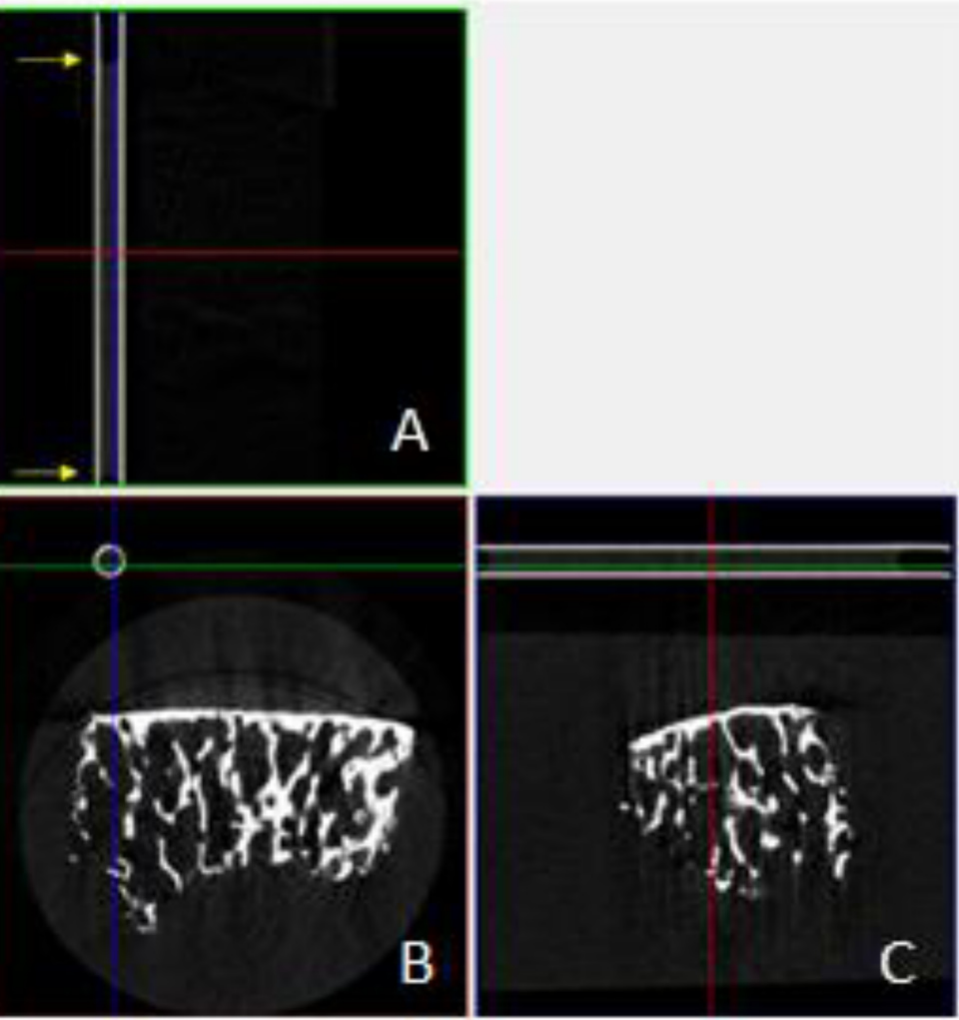
An example of aligning a micro-computed tomography scan of the K-F1 sample with the capillary wall (two parallel white lines), shown in the coronal (**A**), transaxial (**B**), and sagittal (**C**) planes. Blue and green lines, corresponding to x- and y-axis, are parallel to the capillary wall at top left and bottom right images, respectively. Yellow arrows point to the water level in the capillary.

Therefore, to ensure a correct alignment, we taped a glass capillary filled with water to the outer edge of the tube in the sample area. The capillary was made an integral part of the imaged sample (ie, it could not be moved in relation to the bone tissue sample). Its shape allowed the alignment of micro-MRI and micro-CT scans. To visualize the water column within the capillary, the reconstruction of the micro-CT scans was somewhat overexposed. First, reconstructed images were rotated in 3D space using DataViewer software (v.1.5.6.2, Bruker SkyScan) until sagittal and coronal projections were parallel to the capillary wall (Figure 1). This repositioning ensured the same orientation of the sample as during micro-MRI scanning process. Next, a subset of thus aligned cross-sections, extending over the length of the water column, was saved with the resolution reduced by a factor of five in all dimensions. This resulted in matching in-plane resolutions, with each MRI localizer slice corresponding to eight micro-CT slices.

To enable manual alignment of micro-CT and MRI images, a pair of auxiliary high-resolution localizer scans was included in the MRI scan protocol. It consisted of a sagittal and a coronal Spin-Echo scan with slightly tilted slices, perfectly aligned with the water column in the capillary. The height of the water column was approximately 2 cm, sufficient to extend beyond the sample on both ends. Based on the acquired images, a final axial high-resolution Spin-Echo localizer scan was set up with slices perpendicular to the capillary and the first slice aligned with the front edge of the water column. This axial localizer was used later in the postprocessing to correct the rotational mismatch in the positioning between micro-CT and MRI.

Bone volume and fat-water ratio in the sample were assessed with a pair of matched high-resolution Proton Density Turbo Spin-Echo scans. For the main measurements, sagittal slices were selected and perfectly aligned with the water column in the capillary, with front edge of the slice aligned to the front edge of the water column. The slice package was horizontally centered in the transverse (axial) plane with respect to the 15-mL tube and extended above the tube by 1.8 mm (equivalent to 100 micro-CT pixels, ie, slightly more than the combined thickness of the tube and the capillary). To reduce scan time, field-of-view height was limited to 12 mm, and the signal from the rest of the tube was cancelled with a saturation slice. Scan resolution was set to be an integer multiple of the micro-CT resolution (18 μm isotropic) in all scans, to avoid interpolation when downscaling micro-CT images during alignment. Specifically, in-plane pixel dimensions were set to 90 μm isotropic, while slice thickness and interslice gap were 270 μm and 90 μm, respectively. Therefore, every MRI slice in the main measurements covered 20 micro-CT slices. Detailed scan parameters are presented in Table 2.

**Table 2:** Sequence-specific parameters for magnetic resonance imaging (MRI) measurements of osteochondral samples

	Localizer sagittal/coronal	Localizer axial	Main scan
Sequence type	TurboRARE* T2w	TurboRARE T2w	TurboRARE PDw/PDw-FS
Scan dimension	2D	2D	2D
Field-of-view (mm)/number of slices	24x16/11	16.2x16.2/25	20x12/39
Resolution (μm)	133/133/(500 + 300)	90/90/(360 + 360)	90/90/(270 + 90)
Repetition time/echo time (ms)	2500/25	2500/25	4000/8
Averages	2	2	4
Sequence-specific parameters	RARE: 8 Echo spacing: 8.33 ms	RARE: 8 Echo spacing: 8.33 ms	RARE: 4 Echo spacing: 4.0 ms
Acceleration	None	None	None
Bandwidth (Hz)	40 000	40 000	166 666
Scan time (min)	1.15	1.50	8.48

*TurboRARE – T2-weighted Turbo Rapid Imaging with Refocused Echoes.

**Figure 2:**
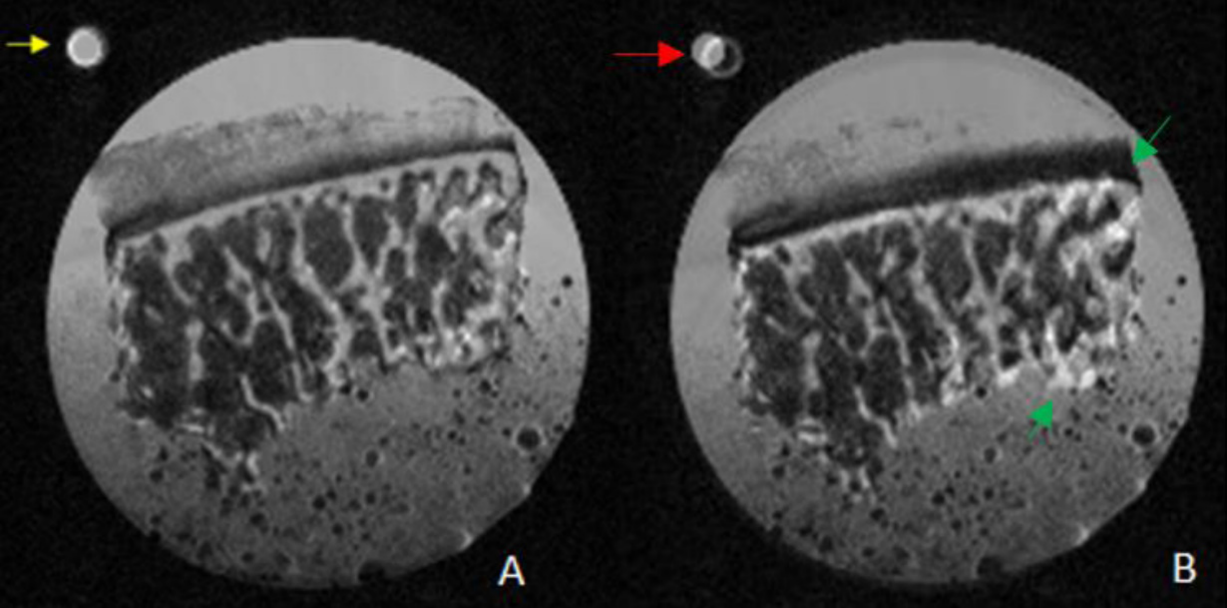
A successful (**A**) and an unsuccessful (**B**) first alignment of a micro-magnetic resonance imaging scan and a micro-computed tomography scan of the K-F1 sample. (**A**) Yellow arrow points to capillaries from both scans, which appear as one single capillary as a result of a successful alignment. (**B**) Red arrow points to capillaries from both scans, which are not aligned. Green arrows point to the parts of the sample that are protruding as a result of an unsuccessful alignment.

Micro-CT and micro-MRI scans were aligned with ImageJ software (v. 1.8.0_172, National Institute of Mental Health, Bethesda, MD, USA) using our in-house Align Images plugin (*https://mef.unizg.hr/znanost/istrazivanje/web-stranice-projekata/projekt-hrzz-hipocart/).* The micro-CT image data set and the localizer micro-MRI image sequence were imported. The localizer image was flipped horizontally, so that in both images the capillary was in the top left corner. Visually identical micro-CT and micro-MRI slices were manually selected and both slices were saved as TIF images in a separate folder. The micro-MRI image was saved as the bottom image and the micro-CT image as the top image. With the Align Images plugin, the selected slices were manually translated and rotated until the top edge of the subchondral bone coincided, and the water inside the capillary (detected by the MRI) fell inside the capillary walls (detected by the micro-CT) (Figure 2). The angle of rotation (N_R_) was written down and reused in DataViewer software (v.1.5.6.2, Bruker SkyScan) to produce sagittal micro-CT slices perfectly matching the MRI sagittal slice orientation.

The micro-CT image data set was then opened in DataViewer software and rotated by the opposite angle of rotation (-N_R_). Volume of interest (VOI) size was defined by the sample’s x- and y-dimensions, and a full z-axis size. The sagittal image data set was saved.

The micro-CT image data set was imported to CTAn software (v. 1.19.11.1, Bruker SkyScan). A ROI of the trabecular bone was manually drawn on the middle slice (N). ROI was selected at the top part of the sample, within the trabecular bone and underneath the subchondral plate. In order to avoid potential infiltration of water into marginal parts of the sample, ROIs were placed at least 0.5 mm from the sample’s edge. More ROIs were then drawn on slices N-20, N-40, N-60, N+20, and N+40. ROI shapes for slices between the manually drawn ones were chosen as “interpolated.” The ROI shape for slices N-61 and N+41 was set to be “empty.” The new data set from ROI was saved and then processed for further micro-MRI analysis using an in-house task list “Save CT ROI binary,” and the results were saved in a separate folder.

**Figure 3:**
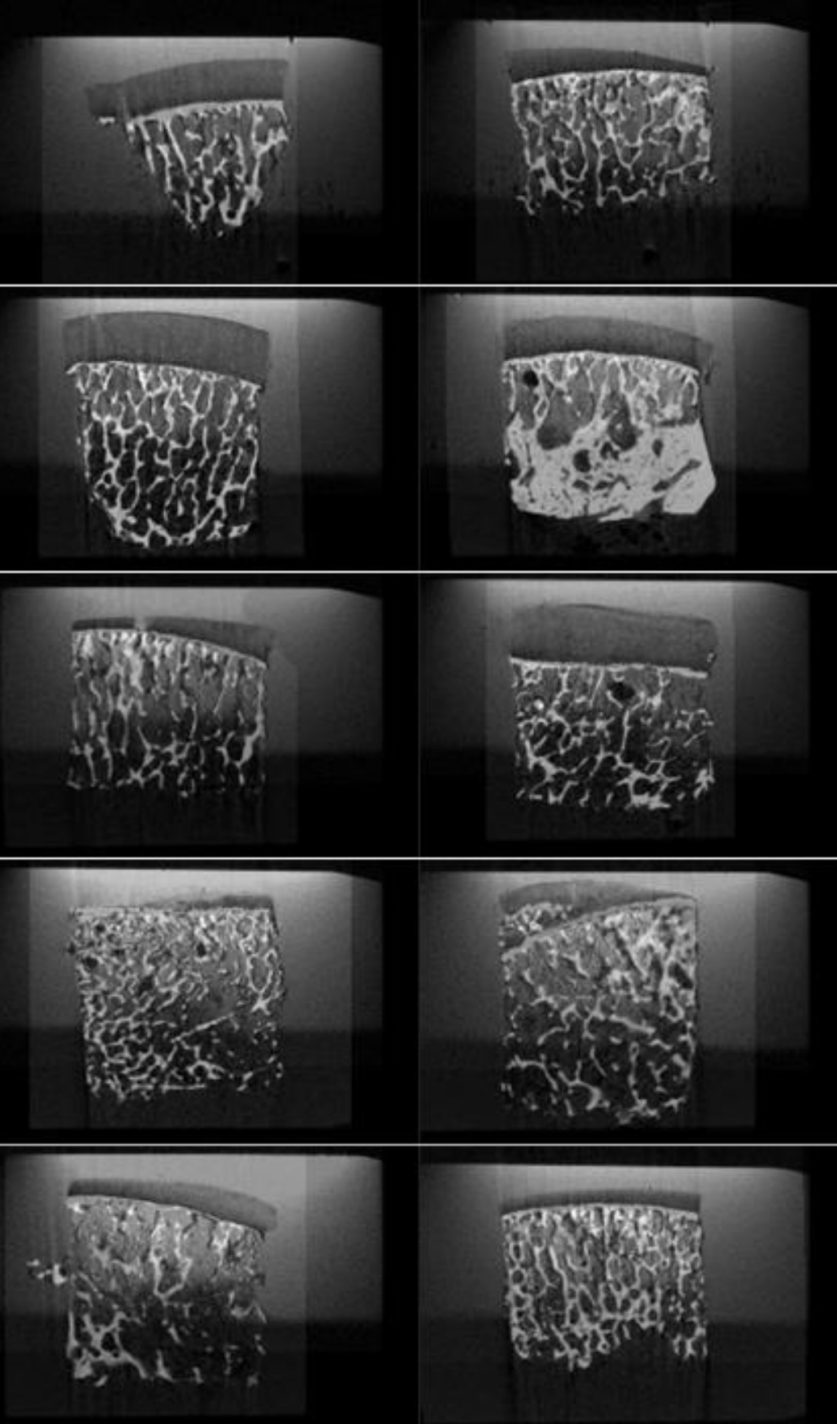
The final alignment of micro-computed tomography and micro-magnetic resonance imaging scans of all 10 samples.

Using an in-house ImageJ “Adapt CT ROI to MRI” plugin, we adapted the position of manually drawn CT ROIs for micro-MRI analysis. The micro-CT image data set and a PDw micro-MRI image were imported. A micro-MRI slice most similar to the middle micro-CT slice was manually selected. Micro-CT and micro-MRI slices were then aligned using our Align Images plugin (Figure 3). Since the scans were already correctly rotated, only the x- and y-coordinates were manually determined and written down. The micro-CT data set was then opened, and the ROI set with position corrected for micro-MRI was saved in a new folder.

### Micro-CT bone volume calculation

Bone volume in total tissue volume (BV/TV) was calculated from micro-CT scans. Step 3b VOI data set was imported into CTAn software. BV/TV was calculated using an in-house task list, with thresholding values for binarization of ROIs set between 75 and 255.

### Statistical analysis

Normality of distribution was tested with the Shapiro-Wilk test. The agreement between micro-CT and micro-MRI analyses was assessed with Bland-Altman analysis. One-sample *t*-test was used to determine whether the differences between the methods significantly differed from the distribution with the mean of 0.

## Results

**Figure 4:**
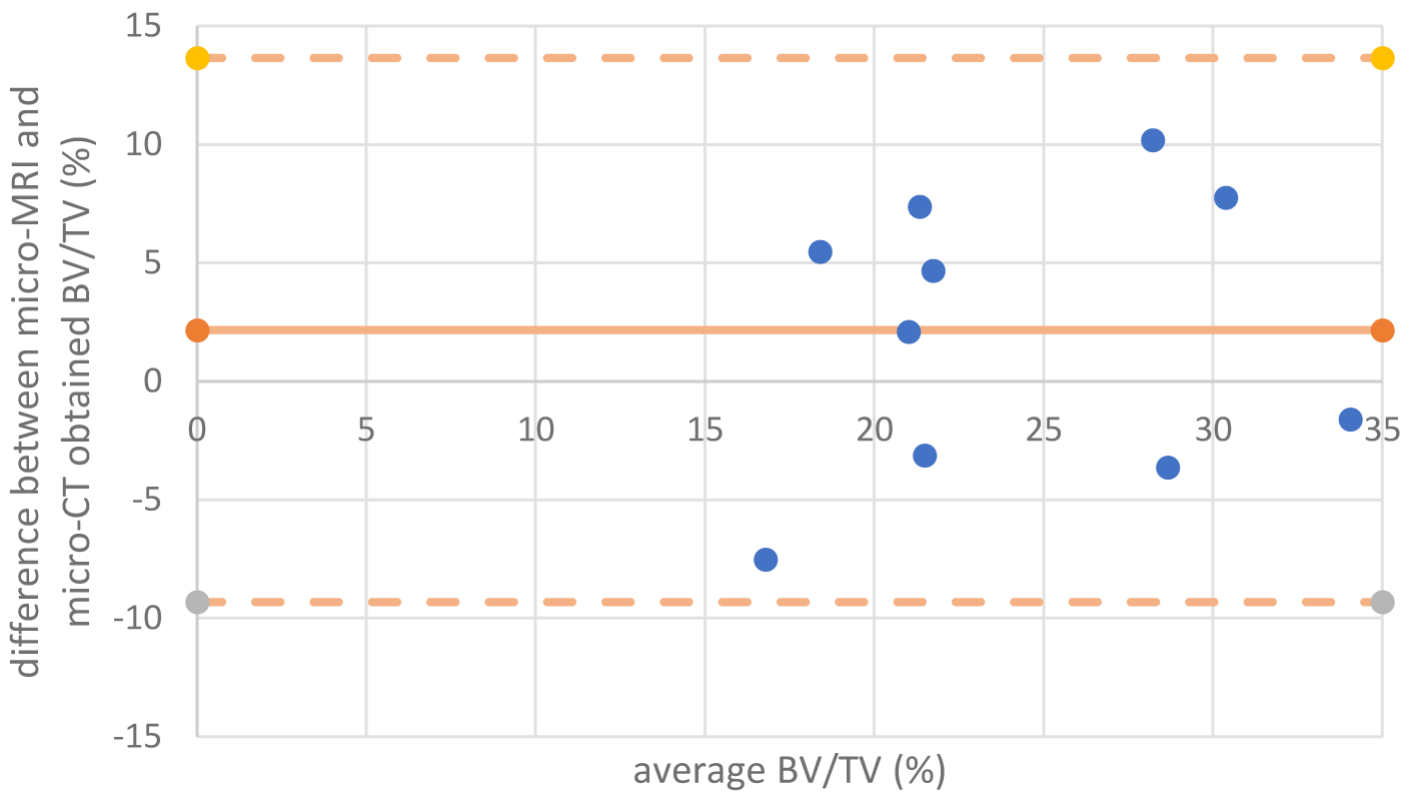
Bland-Altman anaylsis of agreement between micro-computed tomography (CT) and micro-magnetic resonance imaging (MRI) analyses, with the average difference between micro-MRI and micro-CT obtained bone volumes of 2.167%. BVTV – bone volume in total tissue volume.

BV/TV percentages, where tissue volume represents the total volume of a ROI, and fat-to-water ratios of bone marrow calculated from micro-MRI scans are shown in Table 3. The agreement between micro-CT and micro-MRI analyses (Figure 4) was good, with the average difference between bone volumes obtained by micro-MRI and micro-CT of 2.167%. The differences between the two methods were not significant (*P* = 0.272).

**Table 3:** Bone volume in total tissue volume (BV/TV) percentages and fat-to-water ratios of bone marrow calculated from micro-magnetic resonance imaging (MRI) scans

Sample	BV/TV (%)	Fat-to-water ratio
K-F1	30.480	0.693
K-F2	34.856	0.795
K-F3	26.499	0.790
K-F4	15.667	0.710
K-F5	20.539	0.519
K-F6	19.965	0.690
K-F7	19.400	0.368
K-F8	17.659	0.430
K-F9	23.141	0.649
sK-F10	23.043	0.623

*BV/TV, defined as “total volume of binarized objects within the VOI” (31), calculated from micro-computed tomography scans are shown in Table 4.

**Table 4:** Bone volume fractions (BV/TV) calculated from micro-computed tomography (CT) scans

**Sample**	**BV/TV (%)**
K-F1	26.851
K-F2	33.258
K-F3	34.248
K-F4	21.135
K-F5	13.027
K-F6	22.066
K-F7	24.064
K-F8	25.026
K-F9	33.319
K-F10	19.923

## Discussion

Here, we presented a novel method for subchondral bone analysis by micro-MRI based on proton density-weighted images and proton density-weighted images with fat suppression, both of which are commonly used in clinical practice for imaging and evaluation of cartilage (32,33).

Our approach is based on PD weighting, which is preferred to T1 or T2 weighting as it minimizes systemic measurement bias caused by different relaxation rates of fatty tissue and watery tissue (faster T2 relaxation of fat protons causes over-estimation of water content in the voxel). In PDw-FS images, the signal from fat is suppressed, which makes them darker than PDw images of the same tissue (28). The trabecular compartment of the osteochondral unit, the subject of our analysis, consists of bone (ie, the trabeculae) and bone marrow (29). Bone marrow consists mainly of fluid (30) and fats resulting from fat cells accumulation (34). Therefore, in a PDw image of the trabecular compartment, the signal comes from both fat and water protons in the bone marrow, and not from the bone trabeculae. In a PDw-FS image, the signal comes only from water protons. The comparison of these two images provides valuable information on the bone structure. This allowed us to use an original approach to calculate both the bone volume fraction of the trabecular compartment and bone marrow fat fraction based on these two commonly used sequences.

This method of subchondral bone analysis by micro-MRI presents a good alternative to micro-CT. It uses easily available software, provides quantitative information on the structure of the subchondral bone, and strongly positively correlates with the results of micro-CT. Our new method also gives information on the fat composition of the bone marrow. This parameter can be used alongside bone volume fraction to study histological changes associated with OA and other joint disorders.

In our study, we analyzed CT scans using CTAn. It is a software package for Bruker micro-CT, which was used for micro-CT imaging of our samples. Since this software was originally designed for micro-CT scan analysis, we used it for a direct comparison with our results. A great alternative for CT scan analysis is ImageJ plugin BoneJ2, which can determine BV/TV, trabecular thickness/trabecular separation, and a variety of other bone parameters (35).

New methods using MRI have been recently developed for studying the subchondral bone. Most of these methods require clinical MRI machines, ie, machines with magnetic field strengths of 1.5 or 3 T. Some of them have focused on bone marrow fat composition, while others have investigated bone marrow lesions (23,24,36-40). Several protocols have been developed for high-resolution MRI, enabling bone texture analysis, but they do not offer quantitative information about the structure of the subchondral bone, ie, do not accurately quantify bone structural parameters (26,41,42). Compared with these methods, our approach has certain advantages. It provides valuable quantitative information on the structure of the trabecular bone in terms of bone-volume fraction, and takes into account the clinical applicability and availability of MRI scanning protocols. It also assesses bone marrow fat composition in the same analysis, and utilizes free and easy-to-use software. This makes it a user-friendly method that does not necessitate additional expensive software requiring extensive training.

Our study has some limitations. Differences between bone volume fractions obtained from micro-MRI and micro-CT scans can be attributed, to a certain extent, to human error since the images were rotated and aligned by hand. Due to slight deviations during alignment or during the selection of visually identical micro-MRI and micro-CT slices, some of the analyzed ROIs might not have been at identical positions, which possibly led to differences in bone volume fractions. However, even with human error considered, our results show that the proposed new method for analyzing subchondral trabecular bone with micro-MRI highly correlates with micro-CT, the standard method of choice. Another possible limitation for a broad applicability of this method is a limited availability of micro-MRI machines in the laboratory setting.

The presented method can provide valuable information on the structure of the trabecular bone and can be used in OA research as a substitute for micro-CT. Although at this point this is only a proof of concept, with further optimization for clinical MRI machines (1.5, 3, and even 7 T), the presented method has a potential to be used as a diagnostic tool, minimizing exposure to ionizing radiation associated with CT.
